# Adaptive Kalman Filter-Based Single-Beacon Underwater Tracking with Unknown Effective Sound Velocity

**DOI:** 10.3390/s18124339

**Published:** 2018-12-08

**Authors:** Zhong-Chao Deng, Xiang Yu, Hong-De Qin, Zhong-Ben Zhu

**Affiliations:** Science and Technology on Underwater Vehicle Laboratory, Harbin Engineering University, Harbin 150001, China; dengzhongchao@hrbeu.edu.cn (Z.-C.D.); yuxiang332@hrbeu.edu.cn (X.Y.); zhuzhongben@hrbeu.edu.cn (Z.-B.Z.)

**Keywords:** single-beacon underwater tracking, adaptive Kalman filter, extended Kalman filter, effective sound velocity

## Abstract

In the single-beacon underwater tracking system, vehicles rely on slant range measurements from an acoustic beacon to bound errors accumulated by dead reckoning. Ranges are usually obtained based on a presumed known effective sound velocity (ESV). Since the ESV is difficult to determine accurately, traditional methods suffer from large positioning error. By treating the unknown ESV as a state variable, a novel single-beacon tracking model (the so called “5-sv” model) and an extended Kalman filter (EKF)-based solution method have been discussed to solve the problem of ESV estimation. However, due to the uncertainty of underwater acoustic propagation, the probabilistic characteristics of the ESV uncertainty and acoustic measurement noise are unknown and varying both with time and location. EKF, which runs with presupposed noise parameters, cannot describe the practical noise specifications. To overcome the divergence issue of EKF-based single-beacon tracking methods, this paper proposes an adaptive Kalman filter-based single-beacon tracking algorithm which employs the “5-sv” model as the baseline model. Through numerical examples using simulated and field data, both the filter and smoother results show that while implementing the proposed algorithm, the tracking accuracy can be significantly improved, and the estimated noise parameter agrees well with its true value.

## 1. Introduction

Autonomous underwater vehicles (AUVs) have been used for a variety of offshore commercial and scientific applications [1,2,3,4,5,6]. For these applications, accurate tracking of AUVs is essential to ensure the accuracy of the gathered data [7]. Many underwater tracking approaches have been implemented and tested; however, traditional underwater tracking techniques, such as dead reckoning, suffer from unbounded tracking errors. The widely used acoustic approaches, such as long-base line (LBL) system, are restricted by the costly setting up since the location of each deployed beacon in the array must be precisely surveyed before conducting navigation operations. Therefore, tracking AUVs using a single beacon would provide dramatic time and cost savings for underwater vehicle operations, which has been proposed and studied in [7,8,9,10,11,12,13,14,15].

In the single-beacon underwater tracking system, AUVs rely on range measurements from an acoustic beacon with known position to bound errors accumulated by dead reckoning. Early researches on single-beacon tracking can be traced back to Larsen, in which the positioning and velocity error of AUV were treated as state variables, and the extended Kalman filter (EKF) was applied to estimate the vehicle position over time [16]. For positioning an AUV based on time of arrival (TOA) measurements from a single beacon, Casey et al. established a single-beacon tracking model with unknown ocean current using EKF as the state estimator [17]. Later, Fallon developed an AUV- USV (unmanned Surface Vehicle) cooperative tracking model using EKF to update the AUV position [18]. Considering the packet loss problem in underwater communication, Walls proposed a robust origin state method for range-based cooperative tracking based upon a single-beacon tracking scheme, and used the extended information filter (EIF) as the state estimator [19].

In the single-beacon underwater tracking methods mentioned above, the slant range was commonly adopted as the primary measurement, which was obtained from the measured acoustic transit time in conjunction with a presumed effective sound velocity (ESV) for the acoustic propagation between the beacon and the receiver. A known constant ESV was routinely assumed in [8,9,10,11,16,17,18,19]. Since the ESV, in practice, is location dependent, and is very difficult to determine accurately, these methods suffer from large ESV measurement error and consequently has large positioning error. By treating the unknown ESV as a state variable and the transit time as a measurement, Zhu et al. proposed a novel single-beacon localization model (namely the “5-sv” model), and used the EKF as the state estimator [20,21,22]. It was shown that by properly tuning the process and measurement noise parameters, the EKF based on the “5-sv” model could estimate the unknown ESV well, and the tracking performance of the “5-sv” model significantly outperform the traditional model.

By augmenting the unknown ESV as a state variable, the “5-sv” model not only introduced an additional parameter related to the ESV uncertainty needed to be tuned, but also increases the nonlinearity of the measurement model. It had been shown that the EKF performance depended largely on the initial setting of the ESV uncertainty parameter which would stay invariant during the whole estimation process [20]. Furthermore, while implementing the “5-sv” model, the parameters of the process noise covariance matrix and the measurement noise covariance matrix were difficult to tune, and these were usually predetermined based on experience or data post-processing. However, the huge variation of underwater environment leads to a rapid change in the probabilistic properties of the acoustic measurement noise. The problem of EKF estimates degradation occurs frequently while implementing the “5-sv” model due to the violation of known noise statistics assumptions.

To overcome the divergence issue of EKF-based single-beacon tracking methods, this paper will propose an adaptive Kalman filter (AKF)-based single-beacon underwater tracking algorithm. Although the AKF has been intensively investigated to reduce the influence of process noise covariance matrix and measurement noise covariance matrix errors in fields like integrated navigation, economic projection, and chemistry, to the best knowledge of the authors, little literature has been published implementing AKF for single-beacon underwater tracking, especially based on the model with unknown ESV [23,24,25]. Through simulation and field data, the estimation results between using the proposed adaptive algorithm and the traditional EKF will be compared. In addition to the real-time filter tracking, this paper also implements the Rauch-Tung-Striebel (RTS) smoother for post-processing [26,27]. Both simulation and field data will be used to study the possible improvement of the proposed adaptive single-beacon underwater tracking algorithm.

## 2. The “5-sv” Single-Beacon Tracking Model

A review of the “5-sv” single-beacon underwater tracking model, which is served as the baseline model employed in this paper, is offered here as a refresher. This review is also for acquainting the reader with the notation to be used throughout the article.

### 2.1. Kinematic Model

By treating the unknown ESV as a state variable, a single-beacon underwater tracking model—referred to as the “5-sv” (5 state variables) model—was proposed in [20,21,22]:

(1) $$\left\{\begin{array}{c} \dot{x}\left(t\right)\\ \dot{y}\left(t\right)\\ {\dot{v}}_{cx}\left(t\right)\\ {\dot{v}}_{cy}\left(t\right)\\ {\dot{v}}_{e}\left(t\right) \end{array}\right\}=\left\{\begin{array}{c} v_{w}\left(t\right)\cos\varphi \left(t\right)+v_{cx}\left(t\right)\\ v_{w}\left(t\right)\sin\varphi \left(t\right)+v_{cy}\left(t\right)\\ 0\\ 0\\ 0 \end{array}\right\}$$

where the state variables x(t) and y(t) represent the horizontal position of the vehicle; $v_{cx}\left(t\right)$ and $v_{cy}\left(t\right)$ are the two unknown ocean current components in the *x* and *y* directions, respectively; and $v_{e}\left(t\right)$ is the ESV between the beacon and the vehicle. When the ESV is multiplied by the acoustic signal transit time between two underwater point yields the geometric or slant range between them. In Equation (1), $v_{w}\left(t\right)$ and φ(t) are the vehicle speed and heading through water, which are measured from a speed sensor and an electronic compass, respectively.

While treating $v_{w}\left(t\right)$ and φ(t) as control parameters, and including the process noise w(t), Equation (1) can be rewritten as

(2) $$\dot{x}\left(t\right)=F\left(t\right)x\left(t\right)+L\left(t\right)u\left(t\right)+w\left(t\right)$$

where

(3) $$F\left(t\right)=\left[\begin{array}{ccccc} 0 & 0 & 1 & 0 & 0\\ 0 & 0 & 0 & 1 & 0\\ 0 & 0 & 0 & 0 & 0\\ 0 & 0 & 0 & 0 & 0\\ 0 & 0 & 0 & 0 & 0 \end{array}\right],L\left(t\right)=\left[\begin{array}{cc} 1 & 0\\ 0 & 1\\ 0 & 0\\ 0 & 0\\ 0 & 0 \end{array}\right]$$

and

(4) $$x\left(t\right)=\left\{\begin{array}{c} x(t)\\ y(t)\\ v_{cx}\left(t\right)\\ v_{cy}\left(t\right)\\ v_{e}\left(t\right) \end{array}\right\},u\left(t\right)=\left\{\begin{array}{c} v_{wx}\left(t\right)\\ v_{wy}\left(t\right) \end{array}\right\}$$

in which $v_{wx}=v_{w}\cos\varphi$ and $v_{wy}=v_{w}\sin\varphi$ are the in-water speed components of the vehicle in the directions of *x* and *y*, respectively. The process noise vector $w\left(t\right)\in R^{5\times 1}$ accounts for the uncertainty of the kinematic model.

Assuming that the control term u(t) is constant within the discrete sampling interval Δt, we obtain the corresponding discrete kinematic equation

(5) $$x_{k}=A_{k-1}x_{k-1}+B_{k-1}u_{k-1}+w_{k-1}$$

where

(6) $$A_{k}=\left[\begin{array}{ccccc} 1 & 0 & \Delta t & 0 & 0\\ 0 & 1 & 0 & \Delta t & 0\\ 0 & 0 & 1 & 0 & 0\\ 0 & 0 & 0 & 1 & 0\\ 0 & 0 & 0 & 0 & 1 \end{array}\right],B_{k}=\left[\begin{array}{cc} \Delta t & 0\\ 0 & \Delta t\\ 0 & 0\\ 0 & 0\\ 0 & 0 \end{array}\right]$$

and

(7) $$x_{k}=\left\{\begin{array}{c} x_{k}\\ y_{k}\\ v_{cx,k}\\ v_{cy,k}\\ v_{e,k} \end{array}\right\},u_{k}=\left\{\begin{array}{c} v_{wx,k}\\ v_{wy,k} \end{array}\right\},w_{k}=\left\{\begin{array}{c} w_{x,k}\\ w_{y,k}\\ w_{cx,k}\\ w_{cy,k}\\ w_{e,k} \end{array}\right\}$$

in which the subscript *k* denotes the *k*th time step. The uncertainty $w_{k}$ associated with the discrete kinematic model is modeled as Gaussian white noise processes, consisting of five components in three groups: (1) $w_{x,k}$ and $w_{y,k}$, position uncertainty in the *x* and *y* directions; (2) $w_{cx,k}$ and $w_{cy,k}$, ocean current uncertainty in the *x* and *y* directions; and (3) $w_{e,k}$, uncertainty associated with the ESV. The corresponding process noise covariance matrix is:

(8) $$\;Q_{k}\;=\;\left[\;\begin{array}{ccccc} \;\Delta t^{2}\;\left(\;\sigma_{w}^{2}\;\cos^{2}\;\varphi_{k}\;+\;\sigma_{c}^{2}\right)\; & \;\Delta t^{2}\;\sigma_{w}^{2}\;\cos\;\varphi_{k}\;\sin\;\varphi_{k}\;\; & \;0\; & \;0\; & \;0\;\\ \;\Delta t^{2}\;\sigma_{w}^{2}\;\cos\;\varphi_{k}\;\sin\;\varphi_{k}\; & \;\Delta t^{2}\;\left(\;\sigma_{w}^{2}\;\sin^{2}\;\varphi_{k}\;+\;\sigma_{c}^{2}\;\right)\; & \;0\; & \;0\; & \;0\;\\ \;0\; & \;0\; & \;\sigma_{c}^{2}\; & \;0\; & \;0\;\\ \;0\; & \;0\; & \;0\; & \;\sigma_{c}^{2}\; & \;0\;\\ \;0\; & \;0\; & \;0\; & \;0\; & \;\sigma_{e,k}^{2}\; \end{array}\;\right]$$

where $\sigma_{w}$ is the standard deviation of $v_{w,k}$ uncertainty, $\sigma_{c}$ is the standard deviation of $w_{cx,k}$ and $w_{cy,k}$, and $\sigma_{e,k}$ is that of $w_{e,k}$.

### 2.2. Measurement Model

In the application of the Kalman filter for single-beacon underwater tracking, the measurement equation is a nonlinear function of the state variables, with the general form

(9) $$m_{k}=h_{k}\left(x_{k}\right)+v_{k}$$

in which $h_{k}\left(\cdot \right)$ is a nonlinear function. It is assumed that the process and measurement noise $w_{k}$ and $v_{k}$ are mutually independent, zero-mean, Gaussian random processes, with covariance matrices $Q_{k}$ and $R_{k}$, respectively.

In the present application, if the time of emission (TOE) $T_{k}^{e}$ is known and the TOA $T_{k}^{a}$ is measured, then the transit time $T_{k}^{t}$ is given by

(10) $$T_{k}^{t}=T_{k}^{a}-T_{k}^{e}$$

By treating the transit time $T_{k}^{t}$ as the measured quantity for $m_{k}$, we have the nonlinear measurement equation

(11) $$m_{k}=h_{k}\left(x_{k}\right)+\upsilon_{t,k}$$

where $h_{k}$ can be expressed in terms of the state variables $x_{k}$, $y_{k}$ and $v_{e,k}$ to be

(12) $$h_{k}=\frac{\sqrt{{\left(x_{k}-x_{b}\right)}^{2}+{\left(y_{k}-y_{b}\right)}^{2}+{\left(z_{k}-z_{b}\right)}^{2}}}{v_{e,k}}$$

in which $\left(x_{k},y_{k},z_{k}\right)$ and $\left(x_{b},y_{b},z_{b}\right)$ are the positions of the vehicle and the beacon, respectively. Throughout this paper, the vertical position of an underwater vehicle $z_{k}$ assumed to be a known value obtained from a depth sensor. The transit time measurement noise $\upsilon_{t,k}$ is modeled as a white Gaussian noise with variance $R_{t,k}=\sigma_{t,k}^{2}$.

It is also assumed that the vehicle velocity relative to the ground $v_{g}$ is measured from a device, such as a Doppler velocity log (DVL). From the measured $v_{g}$ and the vehicle velocity through water $v_{w}$, using $v_{c}=v_{g}-v_{w}$, one has an indirect measurement for the ocean current:

(13) $$v_{c,k}=\left\{\begin{array}{c} v_{cx,k}\\ v_{cy,k} \end{array}\right\}$$

The corresponding measurement matrix $H_{k}$ associated with the ocean current measurements is simply

(14) $$H_{k}=\left[\begin{array}{ccccc} 0 & 0 & 1 & 0 & 0\\ 0 & 0 & 0 & 1 & 0 \end{array}\right]$$

### 2.3. Comparison with Traditional Model

Traditional single-beacon tracking methods always treated the ESV as a known quantity. In reference to Equations (7) and (8), the corresponding discrete state vector and process uncertainty model are:

(15) $$x_{k}=\left\{\begin{array}{c} x_{k}\\ y_{k}\\ v_{cx,k}\\ v_{cy,k} \end{array}\right\}$$

and

(16) $$\;Q_{k}\;=\;\left[\;\begin{array}{cccc} \;\Delta t^{2}\;\left(\;\sigma_{w}^{2}\;\cos^{2}\;\varphi_{k}\;+\;\sigma_{c}^{2}\right)\; & \;\Delta t^{2}\;\sigma_{w}^{2}\;\cos\;\varphi_{k}\;\sin\;\varphi_{k}\;\; & \;0\; & \;0\;\\ \;\Delta t^{2}\;\sigma_{w}^{2}\;\cos\;\varphi_{k}\;\sin\;\varphi_{k}\; & \;\Delta t^{2}\;\left(\;\sigma_{w}^{2}\;\sin^{2}\;\varphi_{k}\;+\;\sigma_{c}^{2}\;\right)\; & \;0\; & \;0\;\\ \;0\; & \;0\; & \;\sigma_{c}^{2}\; & \;0\;\\ \;0\; & \;0\; & \;0\; & \;\sigma_{c}^{2}\; \end{array}\;\right]$$

From Equations (4), (8), (15) and (16), it is clear that an additional parameter $\sigma_{e}$ is introduced and needed to be properly tuned while implementing EKF based on the “5-sv” model.

Furthermore, when the slant range $r_{k}$ computed from a transit time measurement is used in traditional models, the counterpart of Equation (12) is

(17) $$h_{k}=\sqrt{{\left(x_{k}-x_{b}\right)}^{2}+{\left(y_{k}-y_{b}\right)}^{2}+{\left(z_{k}-z_{b}\right)}^{2}}$$

Interested readers are referred to [20,21,22] for detailed comparison between the “5-sv” model and traditional models.

Compared from Equations (12) and (17), it can be seen that the degree of nonlinearity of the measurement equation in the “5-sv” model is much severer than that of traditional models. In essence, the “5-sv” model has converted the problem of estimating two unknowns ($x_{k}$ and $y_{k}$) from one measurement ($r_{k}$) into the problem of estimating three unknowns ($x_{k}$, $y_{k}$ and $v_{e,k}$) from one measurement ($T_{k}^{t}$). Difficulties of the Kalman tuning process while using the “5-sv” model also appears in that the setting of ESV uncertainty $\sigma_{e}$ interacts with the initial setting of position uncertainties. EKF based on the “5-sv” model is much harder to tune, and more likely to diverge than traditional models if the initial settings are improper. Furthermore, the physics of acoustic wave propagation and the underwater acoustic environment creates some unique differences, including longer transit times and severe refraction through the stratified ocean. Thus, uncertainty associated with the acoustic propagation velocity are very difficult to deal with because they cannot be measured and are generally varying with both space and time. Even if an approximate $\sigma_{e}$ is obtained from experience or data post-processing, divergence issue of EKF based on the “5-sv” model would still occur frequently due to the violation of known noise statistics assumptions. In Section 4.2, a sensitivity study of $\sigma_{e}$ based on filed data will be done to indicate the effect of $\sigma_{e}$ on the estimator performance.

## 3. Windows-Based Adaptive Single-Beacon Tracking Algorithm

Unlike the EKF, the AKF can adjust the noise parameters online based upon the immediate measurements and had proven to be a good solution to improve the robustness of Kalman filters. In this section, a Windows-based adaptive single-beacon tracking algorithm based on the “5-sv” model will be proposed to solve the divergence issue of traditional EKF-based methods.

As presented in many textbooks [26,28], the discrete Kalman filter equations include the prediction and correction equations. Compared with EKFs, the Windows-based AKF introduced an additional step for estimating the process and measurement noise covariance matrices based upon the innovation sequence.

### 3.1. State Prediction

Since the kinematic equation of the “5-sv” model is linear, the prediction equation of the adaptive single-beacon tracking algorithm is the same as the standard Kalman filter, so the estimate of the state vector ${\hat{x}}_{k}^{-}$ and the corresponding covariance matrix $P_{k}^{-}$ are

(18) $${\hat{x}}_{k}^{-}=A_{k-1}{\hat{x}}_{k-1}^{+}+B_{k-1}u_{k-1}$$

and

(19) $$P_{k}^{-}=A_{k-1}P_{k-1}^{+}A_{k-1}^{T}+{\hat{Q}}_{k-1}$$

where ${\hat{Q}}_{k-1}$ is the estimated process noise covariance matrix at the (k−1)th epoch.

Throughout this paper, a variable with a hat, such as $\hat{x}$, represents the estimate of the variable, superscripts “−” and “+” denote quantities associated with *a priori*(prediction) and *a posteriori*(correction) estimates, respectively.

### 3.2. State Correction

For the correction equations of adaptive single-beacon tracking algorithm, the estimate of the state vector ${\hat{x}}_{k}^{+}$ and the corresponding covariance matrix $P_{k}^{+}$ are

(20) $${\hat{x}}_{k}^{+}={\hat{x}}_{k}^{-}+K_{k}\left(m_{k}-h\left({\hat{x}}_{k}^{-}\right)\right)$$

and

(21) $$P_{k}^{+}=\left(I-K_{k}H_{k}\right)P_{k}^{-}$$

in which

(22) $$K_{k}=P_{k}^{-}H_{k}^{T}{\left(H_{k}P_{k}^{-}H_{k}^{T}+{\hat{R}}_{k}\right)}^{-1}$$

is the Kalman gain matrix and ${\hat{R}}_{k}$ is the estimated measurement noise covariance matrix. For the single-beacon underwater tracking algorithm discussed herein, $H_{k}$ is the Jaccbian of nonlinear measurement equation h(•) at the *a priori* estimate ${\hat{x}}_{k}^{-}$

(23) $$H_{k}\left({\hat{x}}_{k}^{-}\right)=\left[\begin{array}{ccccc} \frac{{\hat{x}}_{k}^{-}-x_{b}}{{\hat{v}}_{e,k}^{-}{\hat{r}}_{k}} & \frac{{\hat{y}}_{k}^{-}-y_{b}}{{\hat{v}}_{e,k}^{-}{\hat{r}}_{k}} & 0 & 0 & -\frac{{\hat{r}}_{k}}{{\left({\hat{v}}_{e,k}^{-}\right)}^{2}} \end{array}\right]$$

in which ${\hat{r}}_{k}$ is computed by

(24) $${\hat{r}}_{k}=\sqrt{{\left({\hat{x}}_{k}^{-}-x_{b}\right)}^{2}+{\left({\hat{y}}_{k}^{-}-y_{b}\right)}^{2}+{\left({\hat{z}}_{k}^{-}-z_{b}\right)}^{2}}$$

Noting that ${\hat{z}}_{k}^{-}$ is available from a depth sensor. The measurement innovation $\left(m_{k}-h\left({\hat{x}}_{k}^{-}\right)\right)$ in Equation (20) is denoted by ${\bar{e}}_{k}$ in this paper.

### 3.3. Estimation of Unknown $R_{k}$

From literature [23], the main purpose of the Windows-based adaptive estimation is to estimate the $R_{k}$ through the innovation sequence, in which the innovation is considered to be an intermediate variable. The fundamental principle is making the elements in the actual innovation covariance matrix consistent with their theoretical value. Their actual values are intractable; however, they can be estimated based on maximum likelihood (ML) criterion. The likelihood function is chosen to be the joint conditional probability of measurement given estimated variable within the fixed windows *W*:

(25) $$\begin{array}{cc} J_{k} & =\prod_{j=k-W+1}^{k}p\left(z_{j}|{\bar{C}}_{vj}\right) \end{array}$$

where ${\bar{C}}_{vj}=E\left({\bar{e}}_{j}{\bar{e}}_{j}^{T}\right)$ is the covariance matrix of innovation vector at time $t_{j}$.

Assuming ${\bar{e}}_{j}$ satisfies zero-mean Gauss distribution, Equation (25) can be written as

(26) $$\begin{array}{cc} J_{k} & =\prod_{j=k-W+1}^{k}N\left(z_{j}:h\left({\hat{x}}_{k}^{-}\right),{\bar{C}}_{vj}\right) \end{array}$$

where N(x:μ,Q) means the vector x satisfies Gaussian distribution with mean μ and covariance matrix Q. The probability density function is

(27) $$N\left(x:\mu ,Q\right)=\frac{1}{\sqrt{\left|2\pi Q\right|}}\exp\left(-\frac{1}{2}{\left(x-\mu \right)}^{T}Q^{-1}\left(x-\mu \right)\right)$$

The logarithmic form of Equation (26) is taken as

(28) $$\ln J_{k}=\sum_{j=k-W+1}^{k}-\frac{1}{2}(\ln|2\pi \times {\bar{C}}_{vj}\left|+{\bar{e}}_{j}^{T}{\bar{C}}_{vj}^{-1}{\bar{e}}_{j}\right)$$

To simplify the expression, the following hypothesis is made:

**Hypothesis** **1.** *For a Windows-based AKF, the innovation covariance matrix is constant within the windows width W, that is*

(29) $${\bar{C}}_{vj}={\bar{C}}_{vk},\;\left(k-W+1\right)\leq j\leq k$$

After multiplying Equation (28) by −2 and neglecting the constant term, the ML criterion of maximizing *J* within the windows width *W* becomes the minimization problem which is described as

(30) $$\left\{\begin{array}{c} {\hat{\bar{C}}}_{vk}=\arg\min J_{1,k}\\ J_{1,k}=\sum_{j=k-W+1}^{k}\ln\left|{\bar{C}}_{vk}\right|+\sum_{j=k-W+1}^{k}{\bar{e}}_{j}^{T}{\bar{C}}_{vk}^{-1}{\bar{e}}_{j} \end{array}\right.$$

The minimum value is calculated by the partial of $J_{1,k}$ with respect to ${\bar{C}}_{vk}$

(31) $$\begin{array}{cc} \frac{\partial J_{1,k}}{\partial {\bar{C}}_{vk}} & =W\mathrm{tr}\left\{{\bar{C}}_{vk}^{-1}\right\}-\sum_{j=k-W+1}^{k}{\bar{e}}_{j}^{T}{\bar{C}}_{vk}^{-1}{\bar{C}}_{vk}^{-1}{\bar{e}}_{j} \end{array}$$

To obtain the above formula, the following two relations from matrix differential calculus have been used

(32) $$\frac{\partial \ln\left|A\right|}{\partial x}=\frac{1}{\left|A\right|}\frac{\partial |A|}{\partial x}=\mathrm{tr}\left\{A^{-1}\frac{\partial |A|}{\partial x}\right\}$$

(33) $$\frac{\partial A^{-1}}{\partial x}=-A^{-1}\frac{\partial |A|}{\partial x}A^{-1}$$

Based on the relations from vector inner product $x^{T}x=\mathrm{tr}\left(xx^{T}\right)$ and the trace property of matrix, Equation (31) can be written as

(34) $$\begin{array}{cc} \frac{\partial J_{1,k}}{\partial {\bar{C}}_{vk}} & =\mathrm{tr}\{{\bar{C}}_{vk}^{-1}\left(W{\bar{C}}_{vk}-\sum_{j=k-W+1}^{k}{\bar{e}}_{j}{\bar{e}}_{j}^{T}\right){\bar{C}}_{vk}^{-1}\} \end{array}$$

Let

(35) $$\frac{\partial J_{1,k}}{\partial {\bar{C}}_{vk}}=0$$

then the estimated innovation covariance matrix is obtained as

(36) $${\hat{\bar{C}}}_{vk}=\frac{1}{W}\sum_{j=k-W+1}^{k}{\bar{e}}_{j}{\bar{e}}_{j}^{T}$$

On the other hand, the theoretical innovation covariance matrix can be calculated through the definition of innovation, and have

(37) $${\bar{C}}_{vk}=H_{k}P_{k}^{-}H_{k}^{T}+R_{k}$$

Let the estimated value of the innovation covariance matrix equals to the theoretical value, the estimated $R_{k}$ can then be obtained

(38) $${\hat{R}}_{k}=\frac{1}{W}\sum_{j=k-W+1}^{k}{\bar{e}}_{j}{\bar{e}}_{j}^{T}-H_{k}P_{k}^{-}H_{k}^{T}$$

This estimated $R_{k}$ will be used for calculating the Kalman gain at epoch *k*.

### 3.4. Estimation of Unknown $Q_{k}$

To estimate the unknown $Q_{k}$, rewritten Equation (22) as

(39) $$K_{k}=P_{k}^{-}H_{k}^{T}{\hat{\bar{C}}}_{vk}^{-1}$$

where ${\hat{\bar{C}}}_{vk}$ is assumed to predetermined from Equation (36). Since ${\hat{\bar{C}}}_{vk}$ and $P_{k}^{-}$ are symmetric matrices, Equation (39) can be written as

(40) $${\hat{\bar{C}}}_{vk}K_{k}^{T}=H_{k}P_{k}^{-}$$

Plugging the left side of Equation (40) into Equation (21) yields

(41) $$\begin{array}{c} P_{k}^{-}=K_{k}{\hat{\bar{C}}}_{vk}K_{k}^{T}+P_{k}^{+} \end{array}$$

From Equations (41) and (19), the estimated ${\hat{Q}}_{k-1}$ can be get

(42) $${\hat{Q}}_{k-1}=K_{k}{\hat{\bar{C}}}_{vk}K_{k}^{T}-A_{k-1}P_{k-1}^{+}A_{k-1}^{T}+P_{k}^{+}$$

To calculate ${\hat{Q}}_{k}$, the following hypothesis is made:

**Hypothesis** **2.** *The varying period of $Q_{k}$ is much larger than the sampling period Δt, thus we have*

(43) $$Q_{k}\approx Q_{k-1}$$

Based on the Hypothesis 2, Equations (36) and (42), the estimated $Q_{k}$ at time $t_{k}$ can be obtained

(44) $$\begin{array}{cc} {\hat{Q}}_{k} & =\frac{1}{W}\sum_{j=k-W+1}^{k}K_{k}{\bar{e}}_{j}{\bar{e}}_{j}^{T}K_{k}^{T}-A_{k-1}P_{k-1}^{+}A_{k-1}^{T}+P_{k}^{+} \end{array}$$

Algorithm 1 summarizes the detailed procedure of the Windows-based adaptive single-beacon underwater tracking algorithm.

**Algorithm 1** Windows-based adaptive single-beacon underwater tracking algorithm
**Input:**
${\hat{x}}_{k-1}^{+},P_{k-1}^{+},\left\{{\bar{e}}_{k-W+1},{\bar{e}}_{k-W+2},\cdots {\bar{e}}_{k-1}\right\}$

**Prediction:**
1:
${\hat{x}}_{k}^{-}=A_{k-1}{\hat{x}}_{k-1}^{+}+B_{k-1}u_{k-1}$
2:
$P_{k}^{-}=A_{k-1}P_{k-1}^{+}A_{k-1}^{T}+{\hat{Q}}_{k-1}$


**Correction:**
1:
${\hat{r}}_{k}=\sqrt{{\left({\hat{x}}_{k}^{-}-x_{b}\right)}^{2}+{\left({\hat{y}}_{k}^{-}-y_{b}\right)}^{2}+{\left({\hat{z}}_{k}^{-}-z_{b}\right)}^{2}}$
2:
$H_{k}=\left[\begin{array}{ccccc} \frac{{\hat{x}}_{k}^{-}-x_{b}}{{\hat{v}}_{e,k}^{-}{\hat{r}}_{k}} & \frac{{\hat{y}}_{k}^{-}-y_{b}}{{\hat{v}}_{e,k}^{-}{\hat{r}}_{k}} & 0 & 0 & -\frac{{\hat{r}}_{k}}{{\left({\hat{v}}_{e,k}^{-}\right)}^{2}} \end{array}\right]$
3:
${\bar{e}}_{k}=m_{k}-h\left({\hat{x}}_{k}^{-}\right)$
4:
${\hat{R}}_{k}=\frac{1}{W}\sum_{j=k-W+1}^{k}{\bar{e}}_{j}{\bar{e}}_{j}^{T}-H_{k}P_{k}^{-}H_{k}^{T}$
5:
$K_{k}=P_{k}^{-}H_{k}^{T}{\left(H_{k}P_{k}^{-}H_{k}^{T}+{\hat{R}}_{k}\right)}^{-1}$
6:
${\hat{x}}_{k}^{+}={\hat{x}}_{k}^{-}+K_{k}{\bar{e}}_{k}$
7:
$P_{k}^{+}=\left(I-K_{k}H_{k}\right)P_{k}^{-}$
8:
${\hat{Q}}_{k}=\frac{1}{W}\sum_{j=k-W+1}^{k}K_{k}{\bar{e}}_{j}{\bar{e}}_{j}^{T}K_{k}^{T}-A_{k-1}P_{k-1}^{+}A_{k-1}^{T}+P_{k}^{+}$


**Return:**
${\hat{x}}_{k}^{+},P_{k}^{+},{\bar{e}}_{k}$


## 4. Numerical Studies

Both simulation and field data are used to evaluate the performance of the Windows-based adaptive single-beacon underwater tracking algorithm (to be referred to as the “ASB” algorithm) against that of the EKF-based algorithm (to be referred to as the “ESB” algorithm). In addition to the real-time filter tracking, this paper also implements the RTS smoother for post-processing. The RTS smoother is the most commonly used fixed-interval smoother, which operates backward in time steps after the complete filtering solution has been obtained [26,27].

### 4.1. Simulation Data

**Figure 1:**
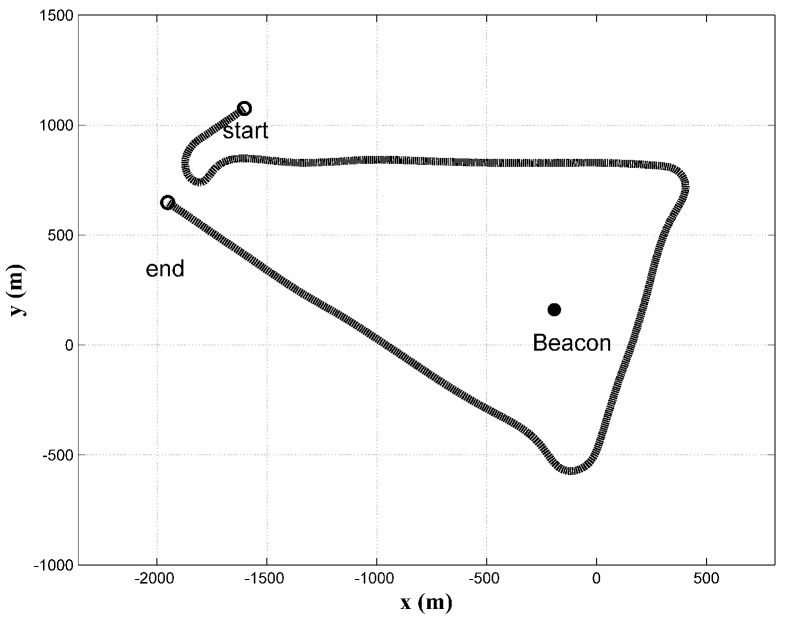
Simulated Trajectory.

The reader is referred to [20] regarding the simulation technique for generating TOA data. For consistency, the trajectory in simulation was chosen to be the same as the trajectory of field data (shown in Figure 1). For the simulation of TOAs, the ESV has been set equal to a constant 1530 m/s during the whole trajectory, with TOEs every 10 seconds. Both ocean current components in the *x* and *y* directions are 0.3 m/s. The process noise parameter $\sigma_{e}$ is chosen as 0.1 m/s. $\sigma_{c}$ and $\sigma_{w}$ are both set to 0.01 m/s. For clarity, the estimation performance of the unknown $R_{k}$ and $Q_{k}$ are studied in two distinct scenarios, respectively. The $Q_{k}$ is assumed to be precisely available during the estimation of unknown $R_{k}$, and vice versa.

The sampling frequency of each simulated measurement and the corresponding noise level characterized by its standard deviation are summarized in Table 1. Notice that the sampling interval of the hydrophone is irregular due to varying acoustic propagation times because of vehicle motion.

In the application of the ASB and the ESB algorithms, the following initial settings have been chosen: (1) a total of 10 meters in both the *x* and *y* directions for the initial position offset; (2) $v_{e,0}=1540$ m/s for the initial ESV; (3) 0.35 m/s for the initial ocean current in both *x* and *y* directions; and (4) $\sigma_{w}=0.01$ m/s; (5) $\sigma_{c}=0.01$ m/s. Specifically, the innovation window width *W* is chosen as 10 while implementing the ASB algorithm.

Fifty Monte Carlo simulations have been done to compare the performance of the ESB and the ASB algorithm. The root mean square (RMS) of the horizontal distance error $\Delta_{H}$ and the estimation error of the ESV $\Delta_{v_{e}}$ are chosen as the evaluation indexes of the Monte Carlo simulations, which are defined respectively as:

(45) $$\left\{\begin{array}{c} {\mathrm{RMS}}_{\Delta_{H}}≜\sqrt{\frac{1}{M}\sum_{s=1}^{M}\left({\left(x_{k}^{s}-{\hat{x}}_{k}^{s}\right)}^{2}+{\left(y_{k}^{s}-{\hat{y}}_{k}^{s}\right)}^{2}\right)}\\ {\mathrm{RMS}}_{\Delta_{v_{e}}}≜\sqrt{\frac{1}{M}\sum_{s=1}^{M}\left({\left(v_{e,k}^{s}-{\hat{v}}_{e,k}^{s}\right)}^{2}\right)} \end{array}\right.$$

in which $\left(x_{k}^{s},y_{k}^{s}\right)$ and $\left({\hat{x}}_{k}^{s},{\hat{y}}_{k}^{s}\right)$ represent respectively the target and the estimated positions at the *s*th Monte Carlo run, and $v_{e,k}^{s}$, ${\hat{v}}_{e,k}^{s}$ represent respectively the target and the estimated ESV at the *s*th Monte Carlo run. *M* = 50 represents the total number of Monte Carlo runs.

#### 4.1.1. Estimate the Unknown $R_{k}$

For the first simulation, the measurement noise parameter is unknown to both the ASB and ESB algorithms, while the parameters of $Q_{k}$ are chosen exactly their target value. Specifically, the initial $\sigma_{e}$ is chosen the same as its target value as 0.1 m/s, while the initial $\sigma_{t}$ is set as 0.05 s with the same initial offset for both the ASB and ESB algorithms.

**Figure 2:**
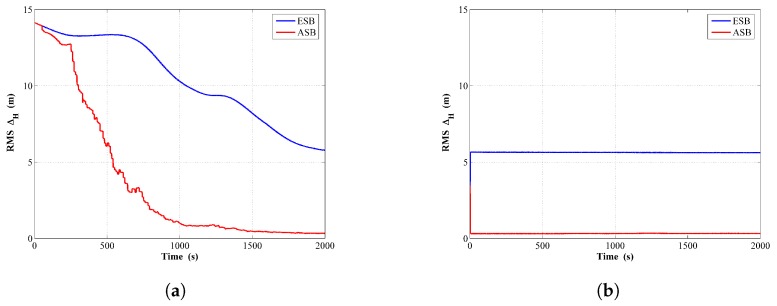
RMS of Horizontal distance error comparison between the ESB and the ASB algorithms using simulation data with unknown $R_{k}$: (**a**) Filter; (**b**) RTS smoother.

The comparison of the ${\mathrm{RMS}}_{\Delta_{H}}$ between the ASB and the ESB algorithm is shown in Figure 2a,b from running the filter and the RTS smoother, respectively. It can be concluded that the tracking error associated with the ASB algorithm is noticeably smaller than that of the ESB algorithm.

**Figure 3:**
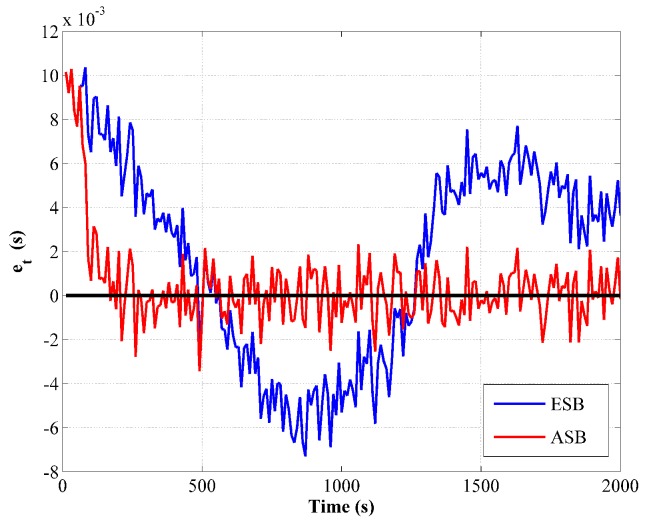
Comparison of transit time measurement innovation between the ESB and the ASB algorithms using simulation data with unknown $R_{k}$.

Furthermore, the innovation of the transit time measurement ${\bar{e}}_{t}$ for the ESB and the ASB algorithm is compared in Figure 3. Since ${\bar{e}}_{t}$ is a signed variable, it is compared from one randomly selected Monte Carlo simulation. From Figure 3, it is obviously that ${\bar{e}}_{t}$ of the ASB algorithm converges rapidly then fluctuates around zero indicating a consistent filter, while that of the ESB algorithm has a large variation due to the improper setting of $\sigma_{t}$.

**Figure 4:**
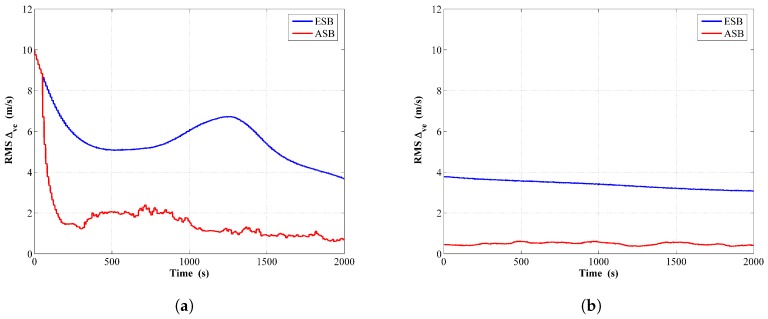
RMS of ESV estimated error by the ESB and the ASB algorithms using simulation data with unknown $R_{k}$: (**a**) Filter; (**b**) RTS smoother.

From the comparison of ${\mathrm{RMS}}_{\Delta_{v_{e}}}$ shown in Figure 4, it can be seen that considering both the converging rate and steady state estimation error, the estimation performance of the ESV while implementing the ASB algorithm outperforms that of the ESB algorithm significantly.

**Figure 5:**
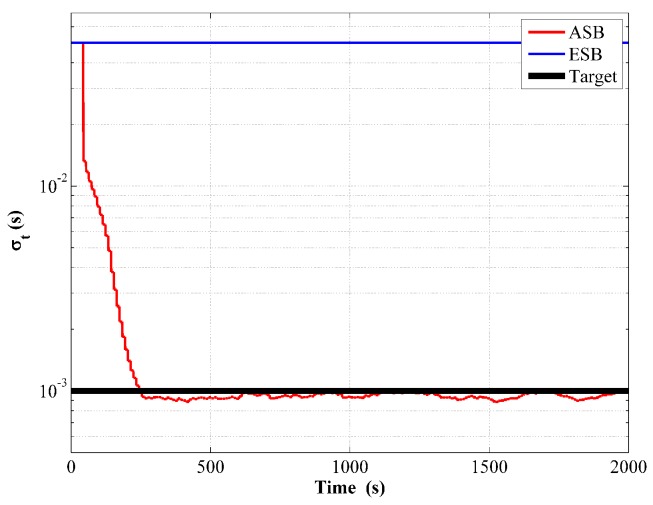
Estimated measurement noise of the ESB and the ASB algorithms using simulation data with unknown $R_{k}$.

Finally, the mean value of the estimated $\sigma_{t}$ while implementing the ASB algorithm from running 50 Monte Carlo simulations is shown in Figure 5. The estimated $\sigma_{t}$ agrees well with its target value, while that in the ESB algorithm remains invariant as respected.

#### 4.1.2. Estimate the Unknown $Q_{k}$

In the second scenario, the process noise parameter is unknown to both the ASB and ESB algorithms, while the parameter of measurement noise is chosen exactly their target value. Specifically, the initial $\sigma_{e}$ is set as 0.5 m/s for both the ASB and ESB algorithms with the same initial offset. To eliminate the possible impact of measurement noise, the hydrophone is kept clean without random noise contamination while generating the simulation data, and the initial $\sigma_{t}$ is chosen as zero for consistency.

**Figure 6:**
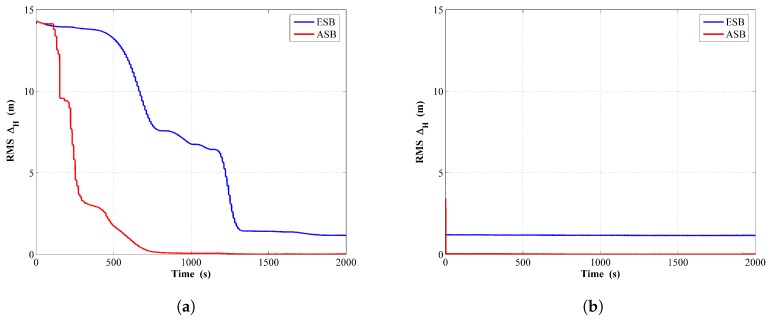
RMS of horizontal distance error comparison between the ESB and the ASB algorithms using simulation data with unknown $Q_{k}$: (**a**) Filter; (**b**) RTS smoother.

For comparing the performance of the ASB algorithm with those of the ESB algorithm, the ${\mathrm{RMS}}_{\Delta_{H}}$ for both two algorithms are shown in Figure 6a,b from running the filter and the RTS smoother, respectively. Clearly, using the ASB algorithm can significantly enhance the tracking accuracy over the ESB algorithm.

**Figure 7:**
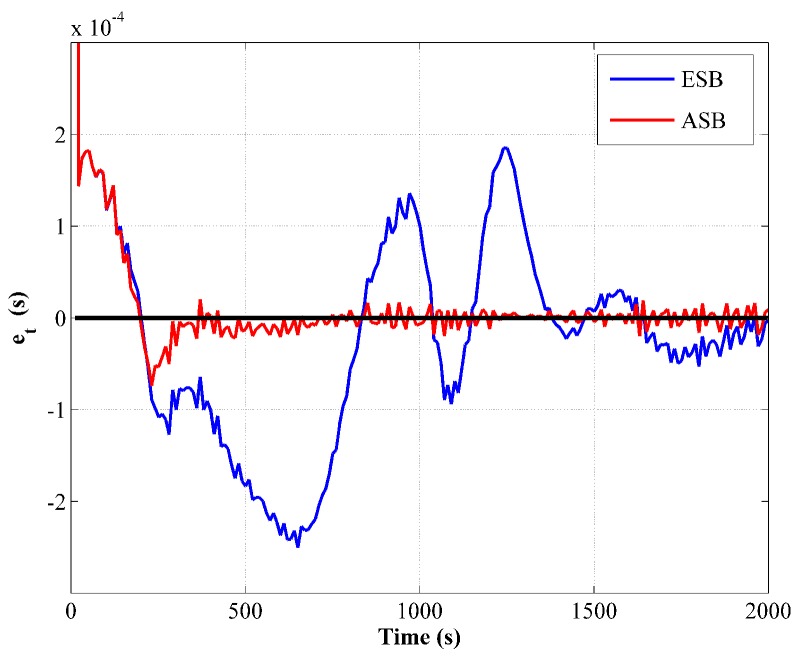
Comparison of transit time measurement innovation between the ESB and the ASB algorithms using simulation data with unknown $Q_{k}$.

The comparison of the measurement innovations is shown in Figure 7, in which similar features with the unknown $R_{k}$ scenario can be observed.

**Figure 8:**
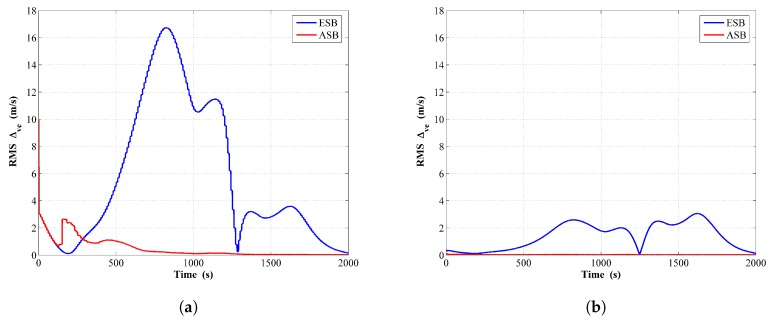
RMS of the ESV estimation error comparison between the ESB and the ASB algorithms using simulation data with unknown $Q_{k}$: (**a**) Filter; (**b**) RTS smoother.

The estimated ${\mathrm{RMS}}_{\Delta_{v_{e}}}$ is shown in Figure 8a,b from running the filter and the RTS smoother, respectively. Once again, the estimated ESV of ASB algorithm has a faster converging rate and smaller steady state error than that of ESB algorithm.

**Figure 9:**
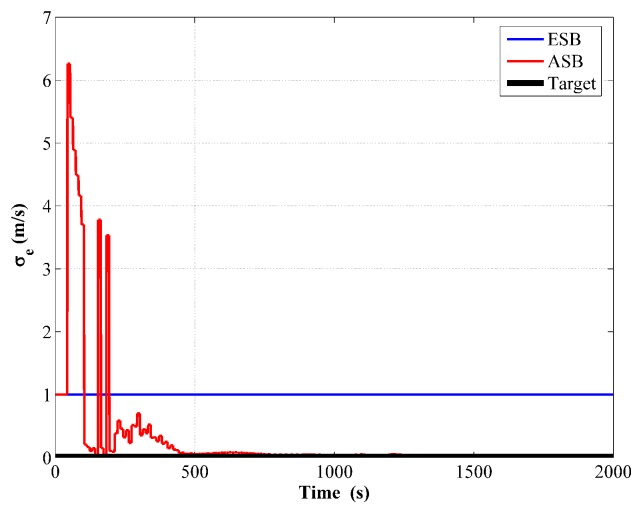
Estimated process noise comparison between the ESB and the ASB algorithms using simulation data with unknown $Q_{k}$.

For completeness, the mean value of the estimated unknown $\sigma_{e}$ from 50 Monte Carlo simulations is shown in Figure 9. Although the estimated $\sigma_{e}$ exist large variation at the beginning, the ASB algorithm can estimate its target value after a certain period of time.

### 4.2. Field Data

The field data used to demonstrate the efficiency of the ASB algorithm was collected from a surface boat equipped with a hydrophone. The boat also had access to the GPS, so the ground truth trajectory of the vehicle was available. A beacon was mounted at the sea floor with a surveyed location. Since the slant range between the vehicle and beacon could be computed from their known locations, the ground truth of every instant ESV could be computed from dividing the slant range by the corresponding transit time, obtained from the TOA measurement minus a known TOE.

**Figure 10:**
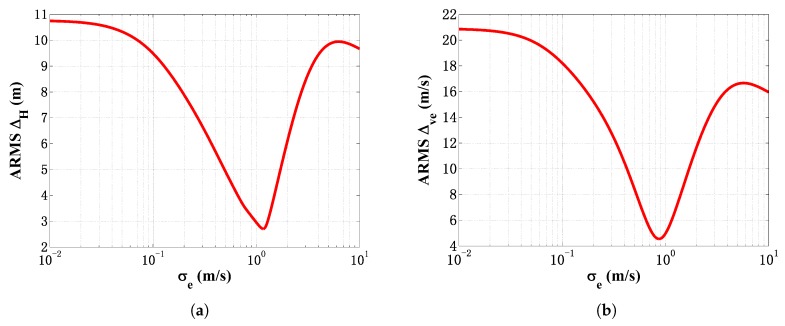
Estimation performance versus $\sigma_{e}$ from EKF using field data: (**a**) ${\mathrm{RMS}}_{\Delta_{r}}$; (**b**) ${\mathrm{RMS}}_{\Delta_{ve}}$.

While implementing the filter and the RTS smoother on the field data, the following initial quantities were chosen: $v_{e,0}=1560$ m/s, $\sigma_{c}=0.01$ m/s, $\sigma_{w}=0.01$ m/s and $\sigma_{t}=0.0001$ s. First, to demonstrate the Kalman tuning issue of traditional ESB algorithms mentioned in Section 2.3, a sensitivity study of $\sigma_{e}$ is carried out based on the field data. Shown in Figure 10a is the averaged RMS of the horizontal distance error ${\mathrm{ARMS}}_{\Delta_{H}}=\sqrt{\sum_{i=1}^{N}\left({\left(x_{i}-{\hat{x}}_{i}\right)}^{2}+{\left(y_{i}-{\hat{y}}_{i}\right)}^{2}\right)}$ with $\sigma_{e}$ varying from 0.01 to 1 m/s. Shown in Figure 10b is the averaged RMS of the estimated ESV error ${\mathrm{ARMS}}_{\Delta_{ve}}=\sqrt{\sum_{i=1}^{K}{\left(v_{e,i}-{\hat{v}}_{e,i}\right)}^{2}}$. *N* and *K* are the total number of fixed sampling interval and transit time measurements, respectively. From Figure 10a,b, it is obviously that $\sigma_{e}$ has a great influence on the tracking performance. Improper $\sigma_{e}$ in the ESB algorithm will degenerate the estimation accuracy drastically.

**Figure 11:**
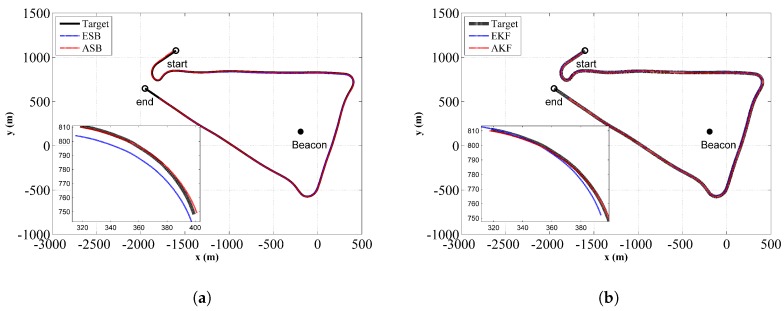
Planar position estimates comparison between the ESB and the ASB algorithms using field data with unknown $Q_{k}$: (**a**) Filter; (**b**) RTS smoother.

**Figure 12:**
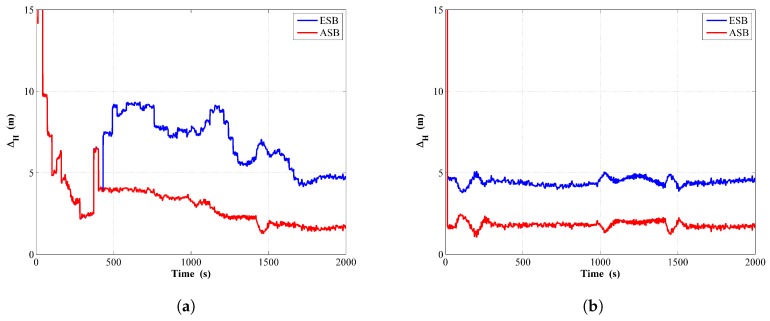
Horizontal distance error comparison between the ESB and the ASB algorithms using field data with unknown $Q_{k}$: (**a**) Filter; (**b**) RTS smoother.

**Figure 13:**
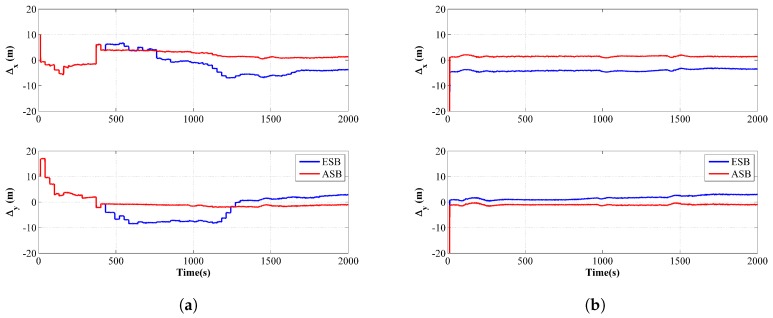
Position component errors of the ESB and the ASB algorithms using field data with unknown $Q_{k}$: (**a**) Filter; (**b**) RTS smoother.

The comparison of the estimated trajectories between the ASB and ESB is shown in Figure 11a,b from running the filter and the RTS smoother, respectively. Similarly, Figure 12a,b are the corresponding comparison of the horizontal distance error $\Delta_{H}$ for the filter and the RTS smoother, respectively. The component errors Δx and Δy for the ASB and ESB are shown in Figure 13. From Figure 11, Figure 12 and Figure 13, it can be concluded that using the ASB could greatly improve the tracking accuracy.

**Figure 14:**
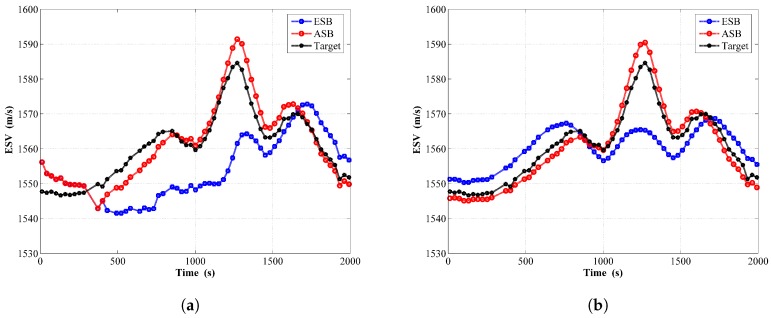
Estimated ESV by the ESB and the ASB algorithms using field data with unknown $Q_{k}$: (**a**) Filter; (**b**) RTS smoother.

Shown in Figure 14 is the estimated ESV from the filter and RTS smoother for these two algorithms, together with the target ESV. Similarly, the estimated ESV while implementing the ASB algorithm agrees better with its target value than the ESB algorithm.

## 5. Conclusions

To eliminate the range measurement error induced by imprecise knowledge of ESV, the so called “5-sv” single-beacon underwater tracking model was recently proposed. However, for applying the EKF based on the specific “5-sv” model, the tracking performance depended largely on the initial knowledge of process and measurement noise statistics, and filter divergence occurred frequently due to the huge variation of underwater acoustic propagation.

To overcome the limitation of EKF-based methods, this paper proposed an adaptive Kalman filter-based single-beacon underwater tracking algorithm basing upon the “5-sv” model. Through numerical examples of using simulated and field data, both the filter and RTS smoother results suggested that the tracking accuracy was significantly improved while implementing the proposed adaptive algorithm. The estimated process and measurement noise parameters also agreed well with their true value.

## Figures and Tables

**Table 1:** Sampling frequency and noise level of simulated measurements.

Sensor	Measurement	Simulated Sampling Frequency (Hz)	Noise (σ)
DVL	$v_{gx},v_{gy}$	1	0.002 m/s
electronic compass	φ	1	0.2${}^{\circ }$
speed sensor	$v_{w}$	1	0.001 m/s
hydrophone	TOA	–	0.001 s
